# “UniCAR”-modified off-the-shelf NK-92 cells for targeting of GD2-expressing tumour cells

**DOI:** 10.1038/s41598-020-59082-4

**Published:** 2020-02-07

**Authors:** Nicola Mitwasi, Anja Feldmann, Claudia Arndt, Stefanie Koristka, Nicole Berndt, Justyna Jureczek, Liliana R. Loureiro, Ralf Bergmann, Domokos Máthé, Nikolett Hegedüs, Tibor Kovács, Congcong Zhang, Pranav Oberoi, Elke Jäger, Barbara Seliger, Claudia Rössig, Achim Temme, Jiri Eitler, Torsten Tonn, Marc Schmitz, Jessica C. Hassel, Dirk Jäger, Winfried S. Wels, Michael Bachmann

**Affiliations:** 1 Helmholtz-Zentrum Dresden-Rossendorf (HZDR), Institute of Radiopharmaceutical Cancer Research, Dresden, Germany; 2 German Cancer Consortium (DKTK), partner site Dresden, and German Cancer Research Center (DKFZ), Heidelberg, Germany; 3 National Center for Tumor Diseases (NCT), University Hospital ‘Carl Gustav Carus’, TU Dresden, Dresden, Germany; 4 Semmelweis University, Department of Biophysics and Radiation Biology, Budapest, Hungary; 5 University of Pannonia, Veszprém, Hungary; 6 Georg-Speyer-Haus, Institute for Tumor Biology and Experimental Therapy, Frankfurt am Main, Germany; 7 German Cancer Consortium (DKTK), partner site Frankfurt/Mainz, and German Cancer Research Center (DKFZ), Heidelberg, Germany; 8 Department of Hematology and Oncology, Krankenhaus Nordwest, Frankfurt am Main, Germany; 9 Institute of Medical Immunology, Martin-Luther-University Halle-Wittenberg, Halle, Germany; 10 Department of Pediatric Hematology and Oncology, University Children´s Hospital Münster, Münster, Germany; 11 Department of Neurosurgery, Section Experimental Neurosurgery and Tumor Immunology, University Hospital ‘Carl Gustav Carus’, TU Dresden, Dresden, Germany; 12 Expermintal Transfusion Medicine, Medical Faculty ‘Carl Gustav Carus’, TU Dresden, Dresden, Germany; 13 Center for Regenerative Therapies Dresden, Dresden, Germany; 14 Institute of Immunology, Medical Faculty ‘Carl Gustav Carus’, TU Dresden, Dresden, Germany; 15 Department of Dermatology and National Center for Tumor Diseases (NCT), University Hospital Heidelberg, Heidelberg, Germany; 16 Department of Medical Oncology, National Center for Tumor Diseases (NCT), University Medical Center Heidelberg, Heidelberg, Germany; 17 Frankfurt Cancer Institute, Goethe University, Frankfurt am Main, Germany; 18 Tumor Immunology, University Cancer Center (UCC) ‘Carl Gustav Carus’, TU Dresden, Dresden, Germany

**Keywords:** Cancer, Immunology, Oncology

## Abstract

Antigen-specific redirection of immune effector cells with chimeric antigen receptors (CARs) demonstrated high therapeutic potential for targeting cancers of different origins. Beside CAR-T cells, natural killer (NK) cells represent promising alternative effectors that can be combined with CAR technology. Unlike T cells, primary NK cells and the NK cell line NK-92 can be applied as allogeneic off-the-shelf products with a reduced risk of toxicities. We previously established a modular universal CAR (UniCAR) platform which consists of UniCAR-expressing immune cells that cannot recognize target antigens directly but are redirected by a tumour-specific target module (TM). The TM contains an antigen-binding moiety fused to a peptide epitope which is recognized by the UniCAR molecule, thereby allowing an on/off switch of CAR activity, and facilitating flexible targeting of various tumour antigens depending on the presence and specificity of the TM. Here, we provide proof of concept that it is feasible to generate a universal off-the-shelf cellular therapeutic based on UniCAR NK-92 cells targeted to tumours expressing the disialoganglioside GD2 by GD2-specific TMs that are either based on an antibody-derived single-chain fragment variable (scFv) or an IgG4 backbone. Redirected UniCAR NK-92 cells induced specific killing of GD2-expressing cells *in vitro* and *in vivo*, associated with enhanced production of interferon-γ. Analysis of radiolabelled proteins demonstrated that the IgG4-based format increased the *in vivo* half-life of the TM markedly in comparison to the scFv-based molecule. In summary, UniCAR NK-92 cells represent a universal off-the-shelf platform that is highly effective and flexible, allowing the use of different TM formats for specific tumour targeting.

## Introduction

Antigen-specific targeting of cancer in a safe and effective manner is challenging since many of the tumour-associated antigens (TAAs) known to date are also expressed to some extent by healthy tissues. The disialoganglioside GD2 is a glycosphingolipid overexpressed by a wide variety of paediatric and adult malignancies including neuroblastoma, melanoma, osteosarcoma, Ewing’s sarcoma, fibrosarcoma and other cancers^1–4^. During foetal development, GD2 plays a role in the developing nervous system^5,6^. Postnatally, its expression is limited to peripheral nerves, certain regions of the central nervous system (CNS), and skin melanocytes^7,8^. Nevertheless, due to its enhanced expression by tumour cells, GD2 provides an interesting target for therapy^9^, and different immunotherapeutic strategies targeting GD2 have already been designed. These include monoclonal antibodies, bispecific antibodies, and chimeric antigen receptor (CAR)-engineered immune cells^10–13^.

CAR-modified lymphocytes represent a promising immunotherapeutic approach that depends on the genetic modification of immune cells to express artificial receptors which bind to specific surface antigens via their extracellular cell-binding domains, and subsequently activate endogenous immune effector mechanisms via their intracellular signalling moieties^14–16^. CAR-engineered T cells have successfully entered clinical practice for the treatment of different B-cell malignancies. Despite the success demonstrated by this technology, it still faces several challenges when targeting solid tumours. These challenges are mainly associated with the inefficient trafficking of CAR T cell into tumours, and the highly immunosuppressive microenvironment which may overcome CAR T cells activation^17^. Other limitations include the finding of appropriate antigens which are not expressed on healthy tissues. Moreover, CAR T cell therapy can lead to several side effects including cytokine release syndrome and neurotoxicities that can be life-threatening if not managed properly^17^. Therefore, novel safety strategies have been developing. Besides T cells, NK cells represent another highly potent effector cell type that can be engineered with CARs. Adoptive transfer of allogeneic NK cells is considered safe, without a high risk of inducing graft-*versus*-host disease (GvHD) as in the case of allogeneic T cells^18^. However, primary NK cells can be challenging to isolate and expand *ex vivo* and may vary in their subset composition and phenotypic characteristics, which can affect their therapeutic activity^19,20^. NK cell lines such as the clinically applicable line NK-92 may provide a valuable alternative to primary NK cells since they can easily be expanded to high numbers and maintained for therapeutic use in the presence of interleukin (IL)-2, while retaining consistent phenotypic and functional features^21,22^. NK-92 cells were initially derived from a non-Hodgkin lymphoma patient, and have similar characteristics to activated peripheral blood NK cells, with the exception of a lack of FcγRIII (CD16) expression^23^. In preclinical studies, NK-92 cells exhibited persistent anti-tumour activity against different hematologic malignancies and some cancers of solid tumour origins^24–26^. In addition, the safety of infusion of irradiated NK-92 cells was demonstrated in early phase clinical trials, with some of the treated cancer patients experiencing long-lasting responses^27–30^. This makes NK-92 cells an interesting option for CAR engineering which provides the cells with antigen-specific targeting, thus further enhancing their anti-tumour activity^31,32^.

We previously described a switchable universal CAR platform termed “UniCAR”, that provides an on/off switch, and thus improved controllability for CAR T cells^33,34^. The UniCAR system consists of two components, one of which is the UniCAR-expressing immune effector cell directed to the peptide epitope E5B9 that is derived from the nuclear antigen La-SS/B^33,35^. As E5B9 is not naturally expressed on the cell surface, a UniCAR effector cell needs to be redirected to the tumour cell by a bispecific second component termed target module (TM). A TM consists of the E5B9 epitope fused to a tumour-specific antigen binding domain, typically a single-chain fragment variable (scFv) of an antibody^36,37^. UniCAR T cells are only active in the presence of a TM. Accordingly, once the respective TM is eliminated, the UniCAR cells are automatically switched off ^36,38^. In addition, high flexibility with respect to the target antigen is achieved by allowing redirection of the same modified T cells to various targets through the simultaneous or sequential use of different TMs. In previous work, we demonstrated highly efficient retargeting of UniCAR T cells to a wide range of antigens, including GD2, CD33, CD123, PSMA, PSCA, STn, EGFR, and others^33,38–41^.

**Figure 1:**
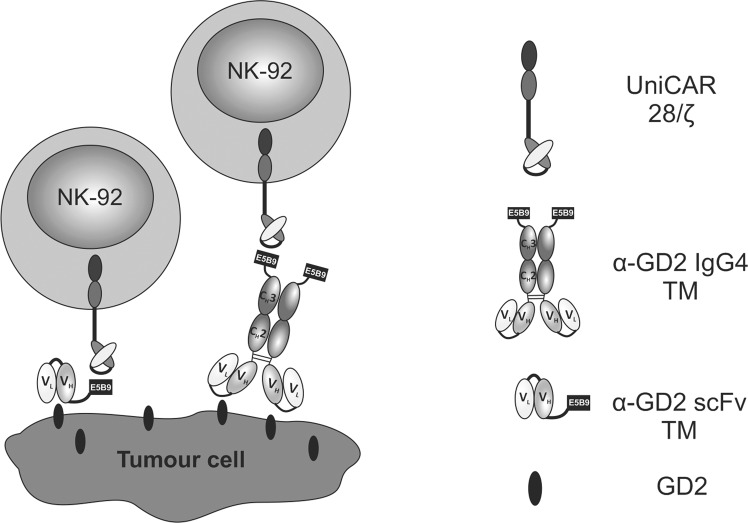
Redirection of UniCAR NK-92 cells towards tumour cells. The UniCAR consists of an extracellular single-chain fragment variable (scFv) antibody directed to the peptide epitope E5B9, the CD28 transmembrane and intracellular costimulatory domain, and the CD3ζ signalling moiety. NK-92 cells modified to express the UniCAR can be redirected to GD2-expressing tumour cells via specific target modules (TMs). These TMs consist of an antibody-based cell-binding domain that recognizes disialoganglioside GD2, and the epitope E5B9 that interacts with the UniCAR molecule. As shown here, different formats of recombinant TMs including scFv-based or human IgG4-based TMs can be used in combination with UniCAR effector cells. V_H_, variable domain of the antibody heavy chain; V_L_, variable domain of the antibody light chain; C_H_, constant domain of the antibody heavy chain (Fc region).

To bring together the advantages of NK-92 cells as an off-the-shelf therapeutic and the versatile UniCAR system, here we generated a stable UniCAR-expressing NK-92 cell line that can be easily maintained and expanded. To test *in vitro* and *in vivo* functionality of these cells, they were combined with a TM selectively recognizing the disialoganglioside GD2. In the case of UniCAR-modified T cells, small antibody derivatives such as a scFv are preferred as a TM to allow rapid clearance from the system in case on-target/off-tumour toxicity occurs. However, this may be less relevant for NK-92 cells which are typically irradiated before application, limiting *in vivo* persistence and preventing expansion in the host^27,28^. Accordingly, in addition to the relatively short-lived scFv-based TM^38^, we also tested a novel homodimeric TM format in which the E5B9 epitope is connected to the GD2-specific antibody domain via an IgG4 Fc region to achieve an extended *in vivo* half-life adapted to the activity half-life of irradiated NK-92 cells (Fig. 1).

## Results

### Modification of NK-92 cells to stably express UniCAR molecules

**Figure 2:**
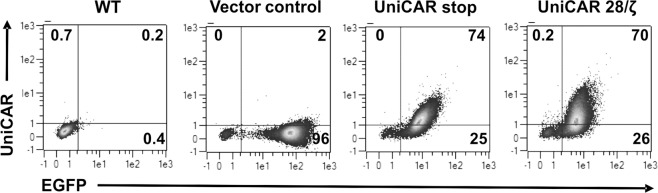
UniCAR expression by engineered NK-92 cells. Transduced NK-92 cells were sorted based on their EGFP expression by flow cytometry. Sorted cells were stained with mAb (clone 7B6) recognizing the E7B6 epitope located in the extracellular domains of UniCAR 28/ζ and UniCAR stop. Binding of mAb 7B6 was detected with PE-conjugated anti-mouse secondary Ab. Numbers on the density plots indicate percentage (%) of positive cells in each gate.

NK-92 cells were modified to express the universal chimeric antigen receptor (UniCAR) by transduction with a lentiviral vector encoding a CAR consisting of an E5B9-specific scFv, a flexible hinge region, the CD28 transmembrane domain and a composite of CD28-CD3ζ signalling domains (UniCAR 28/ζ), followed by an EGFP marker gene (see Methods section). Control cells were transduced with either a similar UniCAR stop construct encoding a truncated UniCAR which lacks intracellular signalling domains or a vector only encoding EGFP (Vector control). Upon transduction, cells were sorted by flow cytometry, yielding highly pure populations of transduced NK-92 cells as judged from their EGFP expression (Fig. 2). Surface expression of the UniCAR molecules on the sorted cells was investigated by staining with an antibody binding to the E7B6 epitope included in UniCAR 28/ζ and UniCAR stop extracellular domains^42^. As expected, UniCAR expression was readily detected in UniCAR 28/ζ and UniCAR stop cells, but not in parental NK-92 (WT) or Vector control cells (Fig. 2).

### Development of GD2-specific scFv- and IgG4-based TMs

**Figure 3:**
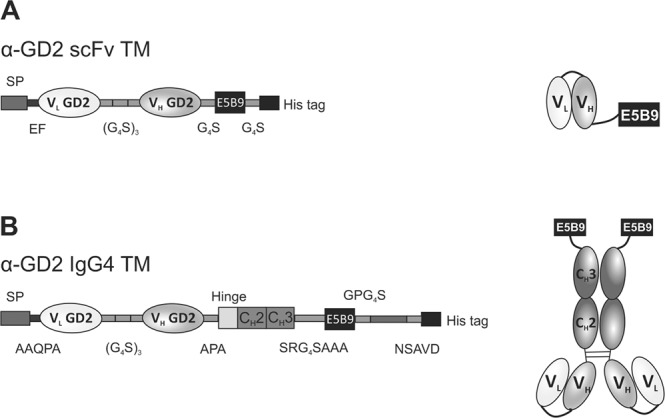
Structure of scFv- and IgG4-based TMs for redirection of UniCAR NK-92 cells. (**A**) The single-chain fragment variable (scFv)-based TM specific for GD2 consists of the variable domains of the antibody heavy (V_H_) and light (V_L_) chains connected to the E5B9 epitope and a polyhistidine tag via glycine-serine linkers. (**B**) To generate an IgG4-based TM, the GD2-specific scFv domain was fused to the hinge, C_H_2 and C_H_3 constant regions of human IgG4, with the E5B9 epitope and a polyhistidine tag placed C-terminally of the C_H_3 region. The amino acid residues forming the peptide linkers between the different domains are indicated. Cysteine residues within the hinge region allow dimerization via disulfide bonds and the formation of a homodimeric IgG4-based TM. SP, signal peptide.

We previously described the generation of the recombinant scFv-based anti-GD2 (α-GD2) TM, which is based on the variable domains of the light and heavy chains (V_L_ and V_H_) of GD2-specific CAR connected by a flexible peptide linker (Fig. 3A**)**^38,43^. This molecule was highly efficient in redirecting UniCAR T cells to GD2-positive target cells^38^. With approximately 30 kDa, the α-GD2 scFv TM is relatively small in size and characterized by a short half-life, which facilitates efficient controllability of UniCAR T cells^36,38^. Hence, *in vivo* application of such a short-lived TM requires continuous infusion to achieve a sufficiently high TM concentration in the blood, and thus in the tumour. However, as NK-92 cells are irradiated before administration into patients, they only have a limited life span^27,44^, which may allow combining UniCAR NK-92 cells with a TM of extended half-life without compromising on safety. Therefore, to test the functionality of a TM with an increased molecular mass, we generated an IgG4-based α-GD2 TM. Inclusion of the IgG4 Fc region and homodimerization of the molecule should result in a longer *in vivo* half-life, which would avoid the need for continuous infusion of the TM.

The specific design of the IgG4-based TM is shown in Fig. 3B. Briefly, a signal peptide (SP) was placed at the N-terminus to allow secretion of the TM. The SP was followed by the GD2-specific scFv, which was further connected to the IgG4 hinge region, and C_H_2 and C_H_3 constant regions through amino acids linkers. The IgG4 Fc region was further linked to the E5B9 peptide epitope, recognized by the UniCAR, and a polyhistidine (6xHis) tag. The IgG4-based molecule is expected to form disulfide-bridged homodimers due to the presence of cysteine residues in the hinge region (Fig. 3B).

### Expression and purification of GD2-specific TMs

HEK 293T cells were used as packaging cells to produce lentiviral particles containing the sequence of the respective TM. Afterwards, the collected viral vectors were used to transduce 3T3 cells and introduce the TM sequences into their genome for stable expression. The TMs were then purified from culture supernatants using Ni-NTA chromatography via the polyhistidine tag (see Methods).

**Figure 4:**
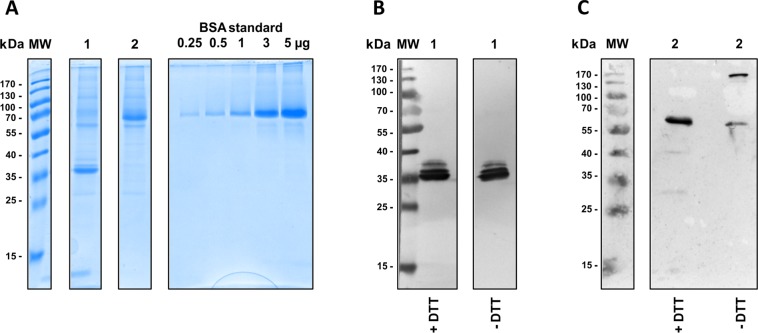
Expression and purification of scFv- and IgG4-based α-GD2 TMs. The TMs were produced as secreted proteins in 3T3 cells which were transduced with lentiviral vectors encoding (1) α-GD2 scFv TM or (2) α-GD2 IgG4 TM. Culture supernatants were subjected to Ni-NTA affinity chromatography, and purified TMs were separated by SDS-PAGE followed by Coomassie staining (**A**), or immunoblot analysis with α-penta-His antibody and alkaline phosphatase-conjugated α-mouse IgG (B + C). Bovine serum albumin (BSA) standards were included in (**A**) to determine protein concentrations. For immunoblot analysis in (**B**) and (**C**), proteins were either separated under reducing (+DTT) or non-reducing (-DTT) conditions. MW, molecular weight marker.

The scFv-based α-GD2 TM has a calculated theoretical molecular mass of around 30 kDa, whereas the monomer of the α-GD2 IgG4 TM is around 56.5 kDa. During protein expression, an IgG4-like homodimer of around 113 kDa is formed. Upon SDS-PAGE and Coomassie staining or immunoblot analysis of purified fractions, the scFv-based α-GD2 TM was only detected as a monomer (Fig. 4A,B), while a large proportion of the IgG4-based TM was indeed found as a homodimer under non-reducing conditions (Fig. 4A,C). The difference in molecular mass between calculated and measured values could be attributable to the glycosylation of the antibody Fc region.

### Binding of GD2-specific TMs to neuroblastoma and UniCAR NK-92 cells

Every part of the designed TM plays a critical role in aspects like the conformation, the binding specificity, or the synapse formation between the engineered immune cells and tumour cells. Therefore, it is important to check the functionality of the different parts of the molecule. The two major components of the α-GD2 TM which are responsible for the linkage between UniCAR NK-92 cells and tumour cells are (I) the scFv part which has affinity for the GD2 antigen on tumour cells, and (II) the E5B9 epitope tag which is recognized by the UniCAR molecules expressed on NK-92 cells.

**Figure 5:**
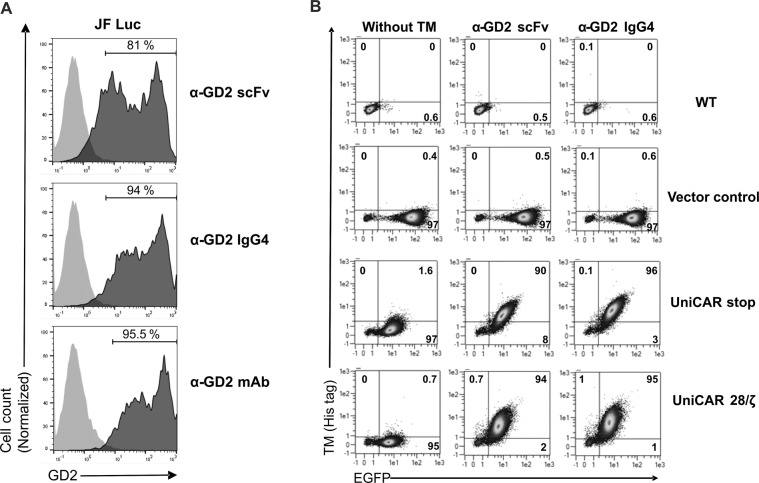
Binding of scFv- and IgG4-based α-GD2 TMs to neuroblastoma cells and UniCAR NK-92 cells. (**A**) JF Luc neuroblastoma cells were incubated with α-GD2 scFv or α-GD2 IgG4 TMs. TM binding to the surface of the cells was then detected with antibody 5B9 specific for the E5B9 epitope tag, followed by Alexa Flour 647-conjugated goat α-mouse antibody (dark grey areas). Control cells were incubated with antibody 5B9 and secondary antibodies in the absence of a TM (light grey areas). In addition, cells were either stained with α-GD2 monoclonal antibody (mAb) or isotype control (bottom panel; dark and light grey areas, respectively). (**B**) To detect binding of the TMs to UniCAR-expressing NK-92 cells, 1.5 × 10^5^ UniCAR 28/ζ and UniCAR stop NK-92 cells were incubated with α-GD2 scFv or α-GD2 IgG4 TMs. TM binding was detected using a PE-conjugated polyhistidine-specific antibody. Parental NK-92 cells (WT) and NK-92 cells transduced with EGFP-encoding vector (Vector control) were included as controls. Numbers shown in the density plots indicate the percentage (%) of positive cells in each gate.

Specific binding of the α-GD2 IgG4 and α-GD2 scFv TMs to GD2-expressing JF Luc neuroblastoma cells was investigated by flow cytometry. Briefly, JF cells were incubated with the TMs, and cell-binding was then detected with an antibody recognizing the E5B9 epitope tag, followed by a fluorescently labelled secondary antibody. Both TMs bound to the surface of JF Luc cells. Staining with α-GD2 scFv TM resulted in 81%, and with α-GD2 IgG4 TM resulted in 94% positive cells, corresponding to 95.5% of positive cells after staining with a GD2-specific commercial antibody included as a positive control **(**Fig. 5A**)**. To investigate specific binding of the TMs to modified NK-92 cells, UniCAR 28/ζ and UniCAR stop NK-92 cells were incubated with α-GD2 scFv or α-GD2 IgG4 TMs. Binding of the TMs was then detected with a fluorescently labelled polyhistidine-specific antibody recognizing the 6xHis tags included in the molecules **(**Fig. 5B**)**. While both α-GD2 TMs strongly bind to UniCAR stop and UniCAR 28/ζ NK-92 cells (90% and 94% positive cells for α-GD2 scFv, and 96% and 95% positive cells for α-GD2 IgG4, respectively), the TMs did not bind to parental and Vector control NK-92 cells as expected. Using an immunofluorescence-based binding assay, we further estimated the apparent K_D_ of the α-GD2 IgG4 to be around 0.2 μM, which is close to the previously published apparent K_D_ value of the α-GD2 scFv TM of 0.3 μM (data not shown)^38^.

### Targeting neuroblastoma cells with UniCAR NK-92 cells redirected by GD2-specific TMs

**Figure 6:**
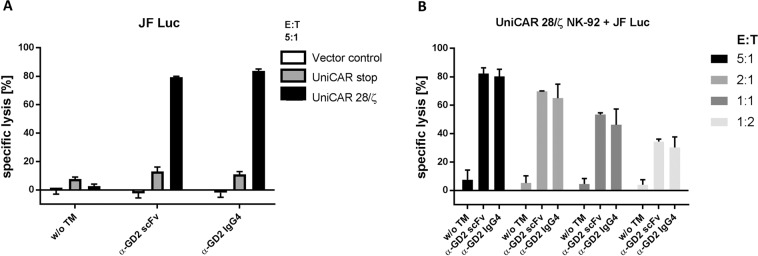
Specific cytotoxicity of UniCAR NK-92 cells redirected by α-GD2 TMs towards neuroblastoma cells. (**A**) UniCAR 28/ζ NK-92 cells were incubated with GD2-positive JF Luc neuroblastoma cells in the absence or presence of α-GD2 scFv or IgG4 TMs at an effector to target (E:T) ratio of 5:1. UniCAR stop and EGFP-expressing NK-92 cells (Vector control) were included as a control. (**B**) UniCAR 28/ζ NK-92 cells were co-cultured with JF Luc cells at different E:T ratios in the presence or absence of the indicated TMs for 4 hrs. Thereafter, specific lysis was measured using a luminescence-based assay. Results are shown as mean ± SD of triplicate samples (**A**) or data from two independent experiments (**B**).

To test the ability of α-GD2 TMs to redirect UniCAR NK-92 cells to GD2-expressing tumour cells, GD2-positive JF Luc neuroblastoma cells were co-cultured with UniCAR 28/ζ NK-92 cells at different effector to target (E:T) ratios in the presence or absence of either the scFv- or IgG4-based TMs at molar concentrations representing comparable numbers of GD2-specific binding moieties and E5B9 epitope tags. EGFP-expressing NK-92 cells (Vector control) or UniCAR stop (lacking signalling domains) were included as controls (Fig. 6A). Cell killing was then evaluated using a luminescence-based cytotoxicity assay (see Methods). As shown in Fig. 6B, UniCAR 28/ζ-expressing NK-92 cells effectively lysed JF Luc cells, which was dependent on the presence of the scFv- or IgG4-based TMs. Tumour cell lysis reached around 80% at an E:T ratio of 5:1, whereas specific lysis of around 30% was seen at an E:T ratio of 1:2 for both TMs. In the absence of a TM, UniCAR 28/ζ NK-92 cells caused only a slight background lysis under the chosen conditions. Likewise, UniCAR stop and Vector control NK-92 cells caused no or only slightly increased background lysis regardless of the presence or absence of the TMs (Fig. 6A).

In conclusion, the UniCAR-expressing NK-92 cells were able to induce antigen-specific tumour cell lysis which was strictly dependent on the presence of α-GD2 TMs.

### Effective working concentrations of anti-GD2 TMs in combination with UniCAR NK-92 cells

**Figure 7:**
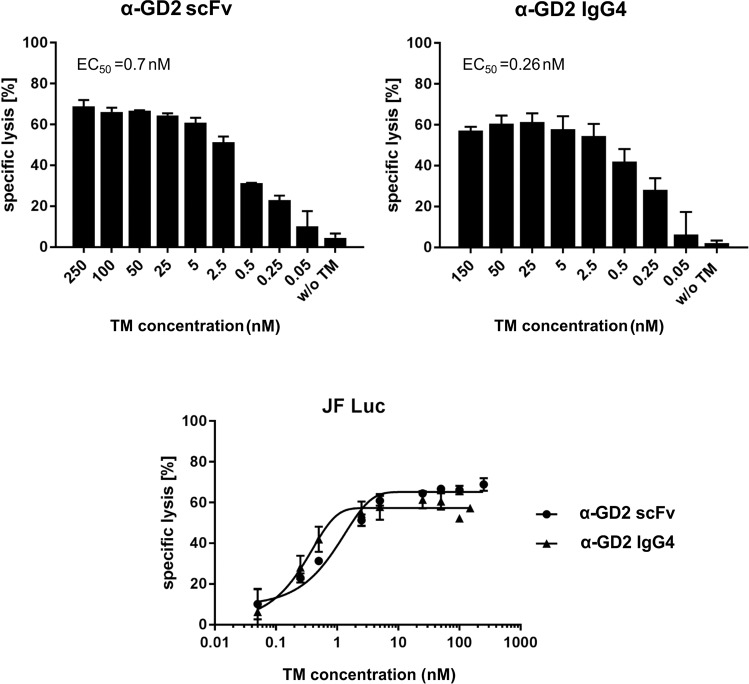
Estimation of the half-maximum effective concentration of α-GD2 TMs. UniCAR 28/ζ NK-92 cells were incubated with GD2-expressing JF Luc cells at an effector to target (E:T) ratio of 5:1. The α-GD2 scFv or α-GD2 IgG4 TMs were added at the indicated concentrations. After 4 hrs of co-culture, target cell lysis was determined using a luminescence-based assay. Results are shown as mean ± SD of data from two independent experiments.

As shown in the previous section, a prerequisite for the antigen-specific killing of tumour cells by UniCAR NK-92 cells is the presence of an appropriate TM. As a consequence, the concentration of the TM plays a critical role in the activation of UniCAR NK-92 cells. Therefore, a range of TM concentrations was tested to determine maximal and minimal TM amounts needed to induce cytotoxicity, as well as estimation of the half-maximum effective concentration (EC_50_). As shown in Fig. 7, target cell lysis increased in a concentration-dependent manner for both TMs, with enhanced cell killing already observed at the lowest concentrations analysed. The calculated EC_50_ value was around 0.7 nM for the α-GD2 scFv and around 0.26 nM for the α-GD2 IgG4 TM.

### Release of interferon-γ from activated UniCAR NK-92 cells

**Figure 8:**
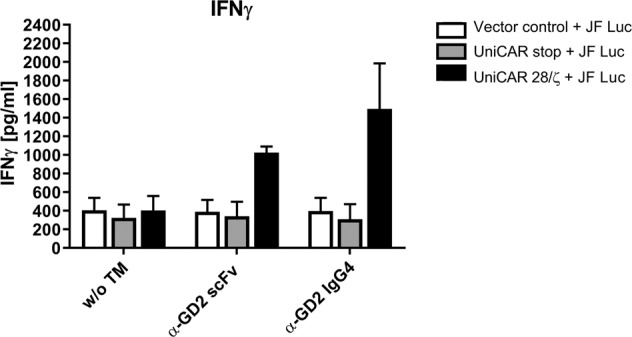
IFNγ release by UniCAR NK-92 cells redirected by α-GD2 TMs. UniCAR 28/ζ NK-92 cells were cultured with JF Luc neuroblastoma cells at an E:T ratio of 5:1 in the presence or absence of α-GD2 scFv or IgG4 TMs. EGFP-expressing (Vector control) or UniCAR stop (lacking signalling domains) NK-92 cells were included as controls. After 4 hrs, supernatants were collected and IFNγ concentrations were determined by ELISA. Results are shown as mean ± SD of two independent experiments.

NK cells are known to produce cytokines upon activation, most prominently (interferon-γ) IFNγ. To investigate the release of IFNγ from UniCAR NK-92 cells upon TM-mediated engagement with tumour cells, JF Luc neuroblastoma cells were incubated with UniCAR 28/ζ NK-92 cells at 5:1 E:T ratio in the absence or presence of the α-GD2 TMs. UniCAR stop or EGFP-expressing NK-92 cells (Vector control) were included as controls. Upon 4 hrs of co-culture, cell-free supernatants were harvested, and the concentration of secreted IFNγ was measured using a conventional ELISA (see Methods) (Fig. 8).

UniCAR 28/ζ-armed NK-92 cells as well as UniCAR stop and Vector control cells secreted comparable basic levels of IFNγ in the presence of tumour cells. However, upon the addition of the α-GD2 scFv or α-GD2 IgG4 TMs, IFNγ levels secreted by UniCAR 28/ζ NK-92 cells increased approximately 2.5- and 3.5-fold. In contrast, this was not the case for NK-92 cells expressing the signalling-deficient UniCAR stop receptor or the EGFP control vector.

### Targeting melanoma cells with UniCAR NK-92 cells redirected by anti-GD2 TMs

**Figure 9:**
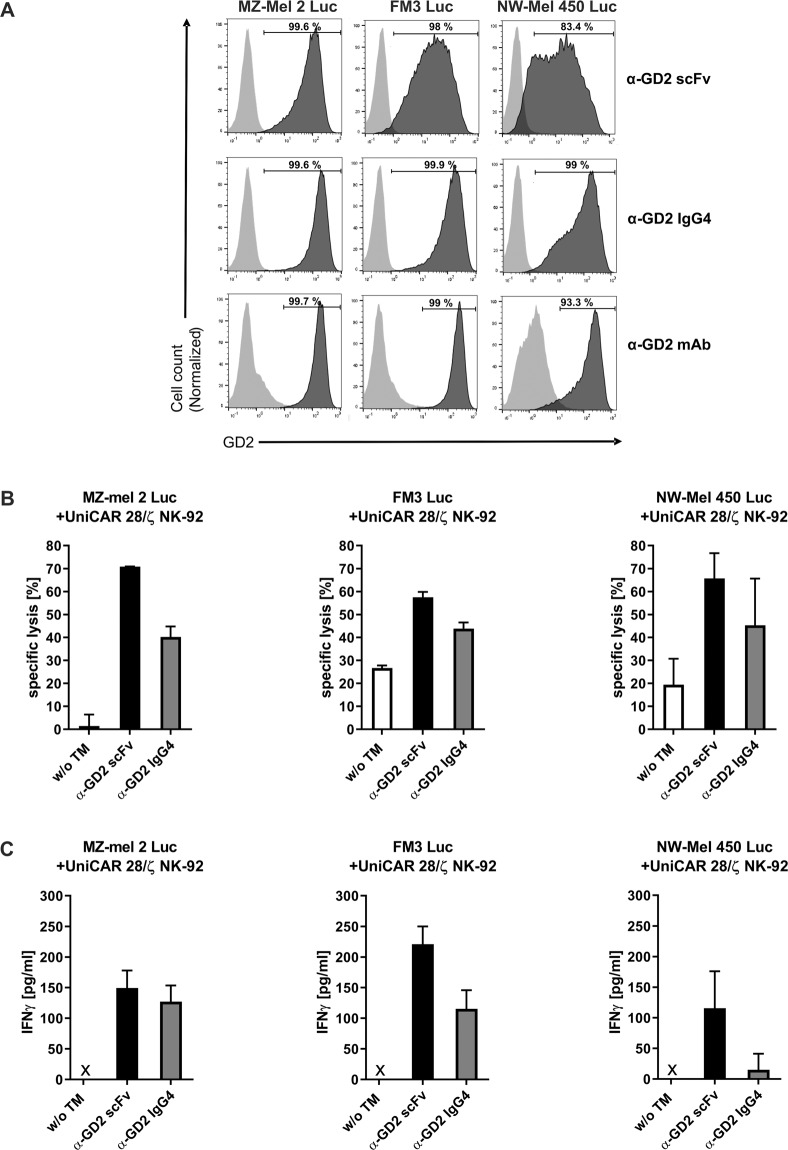
Specific cytotoxicity of UniCAR NK-92 cells redirected by α-GD2 TMs towards melanoma cells. (**A**) MZ-Mel 2 Luc, FM3 Luc and NW-Mel 450 Luc melanoma cells were incubated with α-GD2 scFv or α-GD2 IgG4 TMs. TM binding was then detected with antibody 5B9 specific for the E5B9 epitope tag, followed by Alexa Flour 647-conjugated goat α-mouse antibody (dark grey areas). As controls, cells were incubated with antibody 5B9 and secondary antibody without a TM (light grey areas). To confirm GD2 expression, the melanoma cells were also stained with an α-GD2 monoclonal antibody or an isotype-matched control antibody, followed by Alexa Flour 647-conjugated goat α-mouse antibody (bottom panels; dark grey and light grey areas, respectively). (**B**) UniCAR 28/ζ NK-92 cells were co-cultured at an E:T ratio of 5:1 with the different melanoma cell lines in the presence or absence of α-GD2 scFv or IgG4 TMs for 4 hrs. Thereafter, specific lysis was measured using a luminescence-based assay. Results are shown as mean ± SD of data from two independent experiments. (**C**) Supernatants collected from the cytotoxicity assays were analysed for the presence of IFNγ using an ELISA. Results are shown as mean ± SD for triplicate samples. x, not detectable.

Next, we tested whether this approach can be extended to GD2-expressing tumour targets other than neuroblastoma. First, a panel of melanoma cell lines was analysed for GD2 expression and recognition by the α-GD2 TMs by flow cytometry. The melanoma cells were either stained with an α-GD2 monoclonal antibody, or α-GD2 scFv or IgG4 TMs. All analysed melanoma cell lines expressed GD2, and were recognized by both α-GD2 TMs as indicated by the observed shift of the fluorescence signal as exemplarily shown for MZ-Mel 2, FM3 and NW-Mel 450 melanoma cells in Fig. 9A. More pronounced binding of the IgG4-based TM was observed in comparison to the scFv-based TM.

Then, we investigated the specific cytotoxic activity of UniCAR 28/ζ NK-92 cells against melanoma cells in the absence or presence of the α-GD2 TMs. As shown in Fig. 9B, depending on the individual melanoma cell line tested, UniCAR 28/ζ NK-92 cells displayed a basic degree of cytotoxicity already without the addition of a TM, which likely reflected endogenous natural cytotoxicity of the NK cells. However, addition of either the α-GD2 scFv or IgG4-based TM to co-cultures of UniCAR 28/ζ NK-92 and melanoma cells resulted in a marked increase in tumour cell lysis in comparison to co-cultures without TMs. In contrast, the additions of α-GD2 TMs to co-cultures of UniCAR stop or Vector control NK-92 and melanoma cells did not enhance cell killing (data not shown). To further confirm specific activation of the UniCAR 28/ζ NK-92 cells, supernatants were collected from the cytotoxicity assays and analysed for the presence of IFNγ. Thereby secretion of measurable amounts of IFNγ was only observed when UniCAR 28/ζ NK-92 cells, tumour cells and α-GD2 TMs were combined, whereas no release of IFNγ was found in the absence of TMs (Fig. 9C).

In order to further strengthen the proof of specificity of the α-GD2 TMs, we tested their capability to redirect UniCAR NK-92 cells to the GD2-negative cell line (Panc-89). Neither of the α-GD2 TMs showed any binding to GD2-negative cells nor mediated any killing above the background condition (without a TM). In contrast, the UniCAR NK-92 cells were able to kill the Panc-89 cells in the presence of a TM directed against EGFR which is expressed on the surface of Panc-89 cells (Supplementary Fig. 1A,B).

In conclusion, these data demonstrate that melanoma cells can be specifically targeted and lysed by UniCAR 28/ζ-expressing NK-92 cells in a TM-dependent manner.

### Pharmacokinetic analysis of anti-GD2 TMs in experimental mice

**Figure 10:**
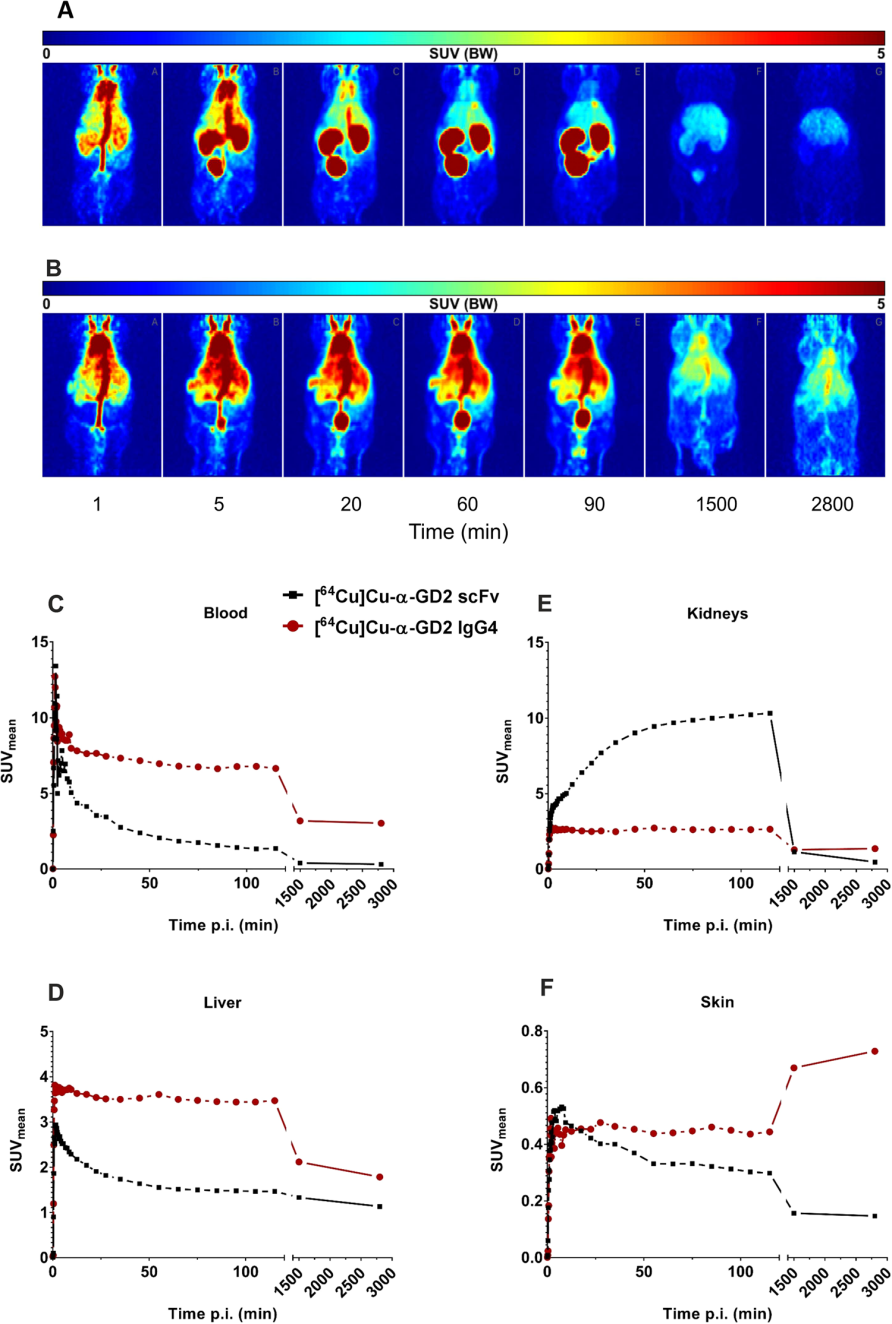
PET analysis of radiolabelled scFv- and IgG4-based α-GD2 TMs *in vivo*. TMs were modified with the chelator NODAGA and radiolabelled with ^64^Cu yielding (**A**) [^64^Cu]Cu-NODAGA-α-GD2 scFv or (**B**) [^64^Cu]Cu-NODAGA-α-GD2 IgG4-based TM, which were intravenously injected into NMRI-Foxn1^nu/nu^ mice. Subsequently, PET scanning was performed at several time points. Coronal sections of the mice are shown. In addition, dynamic PET scans were performed over a period of 50 hrs. Time curves were constructed using mean activity concentration (SUV (g/ml)) in (**C**) blood, (**D**) liver, (**E**) kidneys, and (**F**) skin. SUV, standardized uptake value; p.i., post-injection.

To compare the pharmacokinetic properties of the scFv- and IgG4-based α-GD2 TMs, the recombinant proteins were modified with the chelator NODAGA and then radiolabelled with ^64^Cu. Afterwards, the TMs were intravenously injected into nude NMRI-Foxn1^nu/nu^ mice to allow their detection *in vivo*. Dynamic PET scanning was performed after intravenous injection of the radiolabelled TMs over a period of 50 hrs. Static PET images demonstrated higher enrichment of the α-GD2 IgG4-based TM in the blood for a longer time period when compared to the α-GD2 scFv TM (Fig. 10A,B). PET scanning time curves showed that the half-life of the α-GD2 scFv TM in circulation was around 1.6 hrs, whereas the half-life of the α-GD2 IgG4-based TM was around 39 hrs as estimated from the heart area (Fig. 10C). This difference in the *in vivo* half-life was mainly due to the different elimination routes of the two molecules. Due to its small size, the α-GD2 scFv TM (around 30 kDa) was more enriched in the kidneys and bladder, and as expected could be eliminated much faster through the urinary passage than the larger IgG4-based TM (Fig. 10E). In contrast, the α-GD2 IgG4-based molecule showed higher enrichment in the liver (Fig. 10D). Around 25 hrs post-injection, slightly increased signal of the IgG4-based TM was observed in skin areas, whereas most of the scFv-based TM was already eliminated at this time point (Fig. 10F).

### Anti-tumour effects of UniCAR 28/ζ NK-92 cells in experimental mice

**Figure 11:**
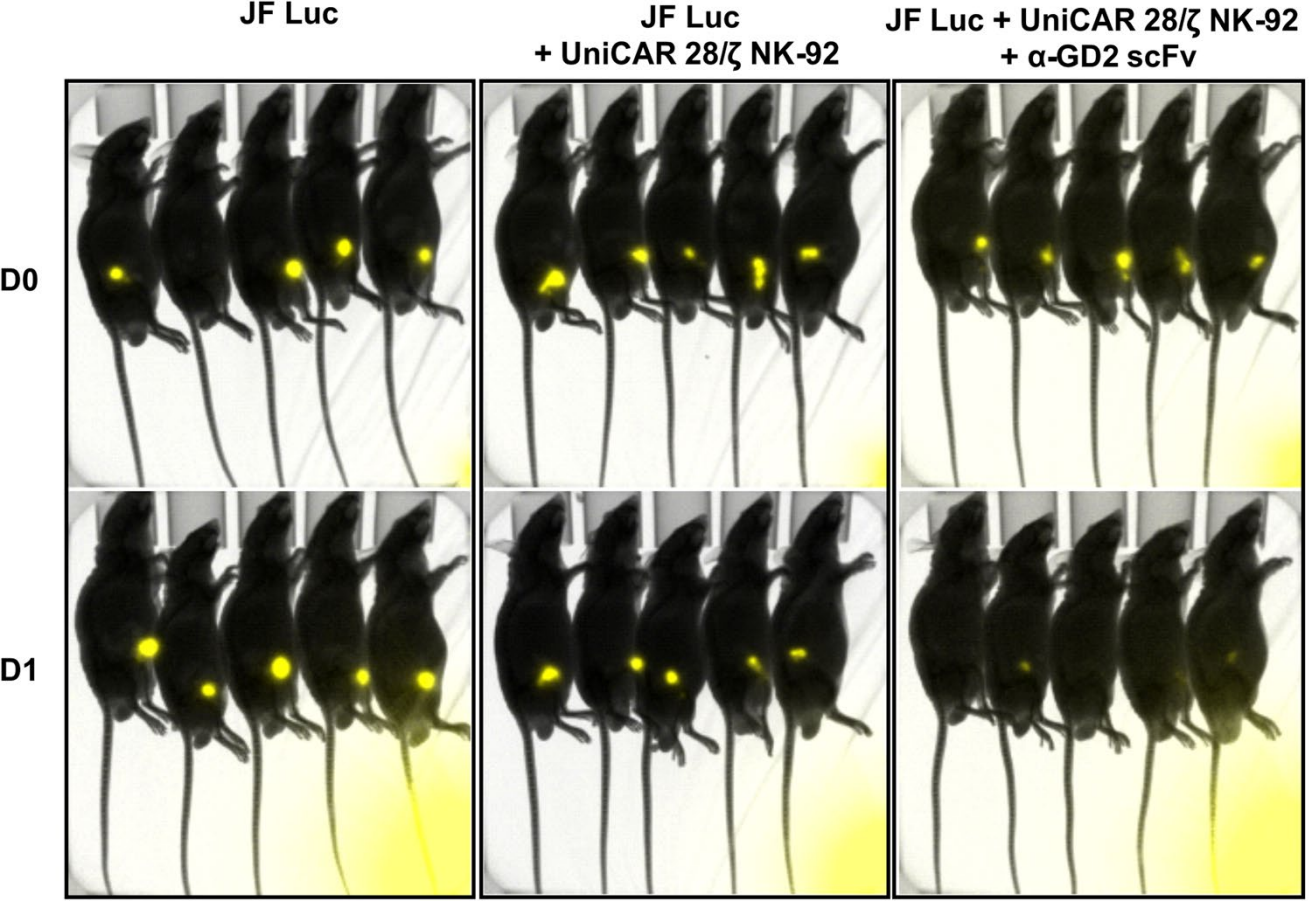
Anti-tumour activity of UniCAR 28/ζ NK-92 cells in experimental mice. Male NMRI-Foxn1^*nu/nu*^ mice were subcutaneously injected with 1 × 10^6^ JF Luc cells expressing firefly luciferase or 1 × 10^6^ JF Luc cells mixed with 0.5 × 10^6^ UniCAR 28/ζ NK-92 cells, or 1 × 10^6^ JF Luc cells mixed with 0.5 × 10^6^ UniCAR 28/ζ NK-92 cells and 6 µg α-GD2 scFv TM (5 animals per group). Bioluminescence imaging was performed on anaesthetized mice after 10 min of i.p. injection of luciferin (15 mg/ml) at day zero (D0) and at day one (D1).

The *in vitro* cytotoxicity experiments described above showed that UniCAR 28/ζ NK-92 cells armed with α-GD2 TMs can selectively kill GD2-expressing neuroblastoma and melanoma cells. To get an insight into the anti-tumour effect of UniCAR 28/ζ NK-92 in the presence of an α-GD2 TM *in vivo*, a co-injection experiment was performed in a JF Luc neuroblastoma xenograft model in nude mice. Three groups of five NMRI-Foxn1^*nu/nu*^ male mice each were subcutaneously injected with JF Luc cells expressing firefly luciferase or JF Luc cells mixed with UniCAR 28/ζ NK-92 cells, or JF Luc cells mixed with UniCAR 28/ζ NK-92 cells and α-GD2 TM, respectively. Subsequently, tumour cell growth was analysed by bioluminescence imaging after intraperitoneal injection of luciferin. As shown in Fig. 11, a marked decrease in the luminescence signal was already observed one day after injection (D1) in the treatment group that had received UniCAR 28/ζ NK-92 cells and the α-GD2 scFv TM, with no visible signal detectable in three mice, and only weak signals found in the other two mice. In contrast, strong luminescence signals representing viable tumour cells were recorded at the same time point in untreated mice and animals that had received UniCAR 28/ζ NK-92 cells without the α-GD2 TM.

## Discussion

The success of CAR-T cells has paved the way for investigating other types of CAR-engineered immune effector cells for the treatment of cancer. Thereby the use of NK cells constitutes a very promising therapeutic approach due to these cells’ natural ability to sense malignant changes in abnormal cells^45^. However, the efficacy of autologous NK cells is limited since their activity can be silenced after encountering self-antigens^21,46^. Accordingly, most current treatment approaches are based on donor-derived allogeneic NK cells or established NK cell lines^32,47^. Here, we employed the human NK-92 cell line due to its unlimited expansion potential and consistent cytotoxicity against cancer cells^21,48^. Preclinical studies have shown the effectiveness of NK-92/engineered NK-92 cells against a variety of cancers including blood malignancies and solid tumours both *in vitro* and *in vivo* models^21,49^. Moreover, enforcing NK-92 cells with CARs has been shown to overcome inhibitory conditions in the tumour microenvironment^32^. The advantageous provided by NK cells in general and by NK-92 cell line, in particular, have made them an attractive option for clinical translation. Currently, several trials employing CAR NK-92 are ongoing, which will provide a valuable insight into the translational value of these cells in the treatment of cancer^49,50^. In this study, NK-92 were modified with universal CAR (UniCAR) molecules in order to create a switchable and highly flexible therapeutic platform^36^. As a target for UniCAR NK-92, GD2 was selected due to its high therapeutic value as it is overexpressed on a variety of tumours, and in case of neuroblastoma it represents one of the very few targets available for immunotherapy^7,9^. In fact, several therapeutic antibodies targeting GD2 were developed, and later the mAb Dinutuximab was approved for patients with high-risk neuroblastoma^3^. Moreover, targeting GD2 with CAR T cells has shown some efficiency in early clinical studies^51^. We have demonstrated previously the efficiency of targeting GD2 with UniCAR T cells^38^. However, T cells can only be used in autologous settings due to the high risk of alloreactivity^52^. Therefore, we investigated in this study the use of UniCAR NK-92 cells as an off-the-shelf therapeutic option for targeting GD2. We confirmed *in vitro* and in experimental mice that NK-92 cells expressing the second generation UniCAR can be redirected to GD2-expressing target cells with both, scFv-based and IgG4-based TMs, leading to effective tumour cell killing in a specific and TM-dependent manner.

NK-92 cells are derived from a non-Hodgkin’s lymphoma patient^23^. Hence, as a safety measure to avoid potential malignant expansion, the cells are usually irradiated before infusion into patients. While this γ-irradiation prevents further proliferation and restricts the life span and cytotoxic activity of NK-92 cells to a few days, it also limits the risk of potential side effects^27^. Consequently, CAR-engineered NK-92 cells are less dependent on a fast safety switch than CAR-T cells in case of unwanted toxicities. There is also no need to actively eliminate CAR NK-92 cells, as their numbers will decrease quickly within few days. Nevertheless, due to the unlimited *in vitro* expansion potential of NK-92 cells and engineered NK-92 variants, multiple dosing is possible, which could at least in part complement the lack of permanent engraftment^30,44^. In the case of UniCAR T cells, the combination with short-lived TMs is preferred since the cells can rapidly expand upon activation in a patient and unwanted side effects may emerge quickly^36^. In contrast, UniCAR NK-92 cells may safely be combined with TMs having a half-life close to the life span of the irradiated effector cells, which eliminates the need for continuous administration of the TM into the patient. Accordingly, we designed a TM for the UniCAR system with an extended half-life, which is based on the structure of human IgG4 combined with the GD2-specific scFv fragment positioned at the N-terminus of the molecule. At the C-terminus, this IgG4-based molecule carries the E5B9 peptide epitope recognized by the UniCAR.

Unlike primary NK cells, NK-92 lack FcγRIII (CD16), and thus cannot be retargeted directly by IgG antibodies^49^. This has prompted the generation of NK-92 cells engineered to ectopically express CD16^53^. While these cells can now trigger ADCC upon interaction with cell-surface-bound IgG molecules, binding of tumour-specific monoclonal antibodies to CD16 can be limited by competition with the patients’ endogenous IgG. Conversely, UniCAR NK-92 cells are activated only by TMs carrying the E5B9 peptide epitope, providing an enhanced level of selectivity and specificity^36,37^. The IgG4 backbone was chosen for the GD2-specific TM because of the weaker capacity of IgG4 to activate complement C1q, and its reduced ability to trigger ADCC when compared to IgG1 and IgG3. This is most likely due to the lower affinity of IgG4 for Fcγ receptors (except for FcγRI)^54,55^.

PET analysis revealed a markedly extended *in vivo* half-life of the IgG4-based TM of around 39 hrs (~1.6 days) in the circulation of mice when compared to that of the scFv-based TM of the same specificity, which was only 1.6 hrs. The longer half-life of the α-GD2 IgG4-based TM is mostly due to the increased molecular mass, which prevents the molecule from being filtered quickly by the kidneys. Moreover, the retention time of these molecules may also be enhanced by recycling through FcRn^55^. After 25 hrs, the radiolabelled IgG4-based TM showed slight signal in the skin areas, which could have been due to binding to Fc receptors on murine immune cells in the dermis (e.g., macrophages and dendritic cells). If needed, such remaining interactions with Fcγ receptors may be reduced by introducing site-directed mutations in the IgG4 constant region^56^. While the half-life of the IgG4-based TM is closer to the life span of irradiated NK-92 cells suggesting this design is well-suited for combination with UniCAR NK-92 cells, the scFv-based construct due to its smaller size could still provide the advantage of faster penetration and more homogenous distribution in the tumour as demonstrated previously^57,58^. Hence, both TM formats may be valuable for therapeutic application depending on the type and stage of the disease as well as overall tumour burden.

Both the scFv- and IgG4-based TMs showed specific binding to GD2-expressing tumour cells but not to the cell line lacking GD2 (see supplementary data). In agreement to previous studies bi-modal expressions can be observed on some of the cell lines, which is due to the heterogeneous expression of GD2 on the cells creating lower and higher GD2-expressing populations^38^. This distribution appears different when stained with the two formats of the TMs, which may be due to the difference in the number of binding sites between the scFv-based (one binding site) and the IgG4-based TM (two binding sites) yielding different avidities.

Here, we showed that UniCAR NK-92 cells specifically and efficiently lysed neuroblastoma and melanoma cells but not the cell line lacking GD2, in the presence of both types of α-GD2 TMs. Moreover, UniCAR NK-92 cells demonstrated a TM dose-dependent effect, meaning that UniCAR NK-92 cells are efficient within a specific window of the TM concentration, which allows further controllability of these cells. In some cases, NK-92 could cause a basic degree of cytotoxicity of melanoma cell lines without the TMs most probably due to the response by natural surface receptors to danger signals on tumour cells^45^. However, the presence of the UniCAR and the TMs had clearly enforced the cytotoxic capability of NK-92 cells. Nevertheless, under the chosen conditions, the α-GD2 IgG4-based TM induced less killing than the α-GD2 scFv in some of the cytotoxicity experiments. This may be due to the increased avidity of the IgG4-based TM, which may stabilize the immunological synapse formation between the effector and target cells, extending the retention time of a UniCAR NK-92 cells at a particular tumour cell and reducing the possibility of serial killing^59^. This was also one of the reasons, why we selected the scFv-based TM for testing of the *in vivo* functionality of UniCAR NK-92. That way we could directly compare the data of UniCAR NK-92 with the previously obtained data of UniCAR T cells^38^.

In addition to TM-mediated lysis of neuroblastoma and melanoma cells, UniCAR NK-92 cells also secreted high concentrations of IFNγ already after 4 hrs of co-culture with target cells in the presence of TMs. This may further enhance therapeutic effects since IFNγ together with other cytokines can synergistically enhance NK-induced lysis by upregulation of adhesion molecules and even GD2 on target cells^60,61^.

Taken together, we demonstrated that the UniCAR system can be readily applied to continuously expanding NK-92 cells, which in combination with GD2-specific TMs efficiently target and lyse GD2-expressing tumour cells such as neuroblastoma and melanoma. Such UniCAR NK-92 cells represent a universal and modular off-the-shelf platform that can be continuously expanded *in vitro*, and also allows the use of different antibody formats as TMs ranging from small scFv fragments to large IgG-based molecules for effective and safe targeting of cancer.

## Methods

### Cell lines

NK-92 cells were cultured in X-VIVO 10 medium (Lonza, Cologne, Germany) supplemented with 5% human plasma (German Red Cross, Dresden) and 500 IU/mL IL-2 (Proleukin S; Novartis Pharmaceuticals, Horsham, UK). The 3T3 cell line used for the production of target modules and HEK 293T human embryonic kidney cells were bought from American Type Culture Collection (ATCC, Manassas, VA, USA). JF neuroblastoma cells (kindly provided by Prof. Malcolm K. Brenner, Houston, TX, USA), FM3, MZ-Mel 2, NW-Mel 450 and Panc-89 cells were transduced with a lentiviral vector for stable expression of firefly luciferase, yielding JF Luc, FM3 Luc, MZ-Mel 2 Luc, NW-Mel 450 Luc and Panc-89 Luc cells as described previously^33,62^^,^. JF, FM3 and Panc-89 cells were cultured in RPMI 1640 medium supplemented with 100 U/ml penicillin and 100 μg/ml streptomycin, 1% non-essential amino acids, 1 mM sodium pyruvate, 2 mM N-acetyl-L-alanyl-L-glutamine and 10% FCS (Biochrom, Berlin). 3T3 and HEK 293T, MZ-Mel 2, NW-Mel 450 cell lines were cultured in DMEM medium supplemented with 100 μg/ml streptomycin and 100 U/ml penicillin, 1% non-essential amino acids and 10% FCS (Biochrom, Berlin). All cells were kept at 37 °C with 5% CO_2_, and cultivated twice every week when they were around 90% confluent.

### Expression and purification of recombinant anti-GD2 target modules

Construction of the α-GD2 scFv TM was described previously^38,43^. To generate the IgG4-based TM, the scFv domain directed against GD2 was first amplified by PCR with the Advantage_HF2 PCR Kit (Clontech Laboratories, Inc., CA, USA) using primer.1 (5′-GGCCCAGCCGGCCGACATCCTGCTGACCC-3′) and primer.2 (5′-CGCCGGCGCGCTGGACACGGTCACG-3′) (Eurofins Genomics GmbH, Germany). The amplified antibody sequence was then cloned into the intermediate plasmid pGEM-T Easy (Promega GmbH, Mannheim, Deutschland), and finally inserted via *Sfi*I and *Mre*I restriction sites into a lentiviral vector p6NST50 containing the hinge and constant region (C_H_2 and C_H_3) sequences of human IgG4 fused to the E5B9 epitope tag and a 6xHis tag. The α-GD2 TMs were expressed in murine 3T3 cells after transduction with lentiviral particles encoding the respective TM sequences. Then, recombinant proteins were purified from cell culture supernatants via Ni-NTA affinity chromatography (Qiagen, Hilden, Germany) facilitated by the C-terminal 6xHis tags included in the molecules, followed by an analysis of identity and yield by SDS-PAGE and immunoblotting as described before^62^.

### Generation of UniCAR vectors

The generation of the UniCAR 28/ζ construct was previously described in detail^33^. Briefly, the UniCAR consists of an extracellular scFv antibody domain directed against the E5B9 epitope, and a hinge region containing a peptide sequence (E7B6) for immunological detection, which like E5B9 is derived from La/SS-B^35^. These sequences are followed by transmembrane, intracellular domains of CD28 signalling and the CD3ζ ITAM motifs. Two additional constructs were included as controls: A Vector control encoding EGFP only, and the UniCAR stop construct which lacks the intracellular signalling domains of CD28 and CD3ζ. The CAR sequences were fused to an EGFP sequence separated by a T2A site derived from the *Thosea asigna* virus to allow the separation of the two proteins during translation by ribosomal skipping^63^.

### Lentiviral transduction of NK-92 cells

NK-92 cells were set at a density of 2 × 10^5^ cells/2 ml in supplemented X-VIVO 10 medium (Lonza Group, Basel) one day before transduction. On the following day, the cells were mixed with lentiviral particles (MOI of 2) together with polybrene (16 μg/2 ml, Sigma-Aldrich Chemie GmbH) and the kinase inhibitor BX795 (8 μM, InvivoGen, France), which had both been shown to improve lentiviral transduction efficiency of NK cells^64,65^. Afterwards, the cells were centrifuged in a 6-well plate for 60 min (1800 × g at 32 °C), followed by 6 hrs of incubation at 37 °C. The transduction mixture was then removed by centrifugation (5 min at 360 × g) and replaced with 2 ml of fresh supplemented X-VIVO 10 medium. On the following day, the NK-92 cells were seeded in 5 ml of medium containing the lentiviral vectors, polybrene and BX795 for 6 hrs at 37 °C. The second transduction mixture was exchanged with fresh medium, and the cells were incubated at 37 °C. Transduction was repeated a third time on the following day. Transduction efficiency was evaluated by flow cytometry detecting EGFP expression, and EGFP-positive cells were isolated by sorting with a FACSAria Fusion flow cytometer (BD Biosciences, Heidelberg, Germany).

### IFNγ-release assay

For detection of IFNγ production, 2.5 × 10^4^ engineered NK-92 cells were seeded in 96-well plates in triplicates together with 5 × 10^3^ target cells. Then 25 nM of α-GD2 IgG4-based TM or 50 nM of α-GD2 scFv TM were added. After 4 hrs, cell-free supernatants were collected and analysed using the OptEIA Human IFNγ ELISA Set (BD Biosciences) according to the manufacturer’s instructions.

### Flow cytometric analysis

For analysis of the binding of TMs to tumour cells, 1 × 10^5^ cells were incubated with 2.5 µg/100 µl of α-GD2 scFv, 4.8 µg/100 µl of α-GD2 IgG4 TM or 2.6 µg/100 α-EGFR TM (yielding an equal molar ratio of binding moieties) for 1 hr, then washed and incubated with 100 µl of a 15 µg/ml solution of the anti-La mAb 5B9 interacting with the E5B9 epitope tag for 30 min. Bound antibody complexes were finally detected with Alexa Flour 647-labelled goat α-mouse IgG (Life Technologies, Thermo Fisher Scientific). For detection of UniCAR surface expression, engineered NK-92 cells were incubated with 100 µl of a 15 µg/ml solution of the anti-La mAb 7B6, and subsequently stained with PE-labelled goat α-mouse antibody (Beckmann Coulter, Krefeld, Germany). Samples were then analysed using a MACSQuant Analyser and MACSQuantify Software (Miltenyi Biotec, Bergisch Gladbach, Germany).

### Luminescence-based cell killing assay

To determine the specific killing of tumour cells, a luminescence-based cytotoxicity assay was used with target tumour cells modified to express Firefly luciferase. For the assays, 5 × 10^3^ tumour cells were co-cultured with engineered NK-92 cells at different effector to target cell (E:T) ratios in the presence of 50 nM of α-GD2 scFv, α-EGFR TM or 25 nM of α-GD2 IgG4 TMs to yield equal ratios of binding moieties. Alternatively, a range of TM concentrations were added in order to determine EC_50_ values. The cells were cultured in 96 well white plates (Chimney Well, Greiner Bio-One GmbH, Germany) in a total volume of 200 μl of supplemented X-VIVO 10 medium per well. Plates were kept at 37 °C in a humidified atmosphere of 5% CO_2_ for 4 hrs. Afterwards, luminescence signals were recorded, and specific lysis was calculated as described previously^38^.

### Antibody conjugation and radiolabelling

Recombinant target modules were modified with the lysine-reactive chelator NODAGA (CheMatech, Dijon, France) and radiolabelled with ^64^Cu as previously described in detail^38^. ^64^Cu was generated at the Helmholtz-Zentrum Dresden Rossendorf (HZDR) with a Cyclotron Cyclone(R) 18/9 via ^64^Ni(p,n) ^64^Cu-nuclear reaction^66^.

### Bioluminescence and immuno-PET imaging of experimental animals

*In vivo* experiments were performed according to the guidelines of the ARRIVE and the European Communities Council Directive (86/609 EEC). All procedures were approved by the Animal Research Ethics Committee of Semmelweis University and the relevant National Authority - the National Authority of Food Chain Safety - (XIV-I-001/29-7/2012, PE/EA/50-2/2019). *In vivo* experiments were conducted with Naval Medical Research Institute (NMRI)-Foxn1^nu/nu^ mice purchased from JANVIER LABS (Saint-Berthevin Cedex, France). To evaluate the anti-tumour activity of UniCAR-expressing NK-92 cells, a co-injection experiment was conducted in 5 weeks old male NMRI-Foxn1^nu/nu^ mice. Three groups, each consisting of five mice were used. The two control groups were subcutaneously injected with either 1 × 10^6^ JF Luc cells alone or in combination with 0.5 × 10^6^ UniCAR 28/ζ NK-92 cells in a total volume of 100 μl of PBS. The treatment group was injected with a mixture of 1 × 10^6^ JF Luc cells, 0.5 × 10^6^ UniCAR 28/ζ NK-92 and 6 μg of the α-GD2 scFv TM in a total volume of 100 μl of PBS. Subsequently, viable tumour cells were detected by bioluminescence imaging combined with X-ray imaging, performed with a Kodak FX PRO imaging device (Kodak Molecular Imaging Systems, New Haven, USA) with 5 min exposure for bioluminescence imaging and 1.3 sec for X-ray imaging. Data evaluation was carried out using the analysis software Bruker MI and Multispectral (Bruker, Karlsruhe, Germany). For pharmacokinetic and distribution analysis of [^64^Cu]Cu-NODAGA-modified TMs, protein amounts corresponding to around 3.7 MBq were injected into the lateral tail vein of NMRI-Foxn1^nu/nu^ mice. This was followed by dynamic PET scans for 50 hrs, and recording of additional static images using a microPET P4 scanner (Siemens Molecular Imaging, Germany). PET data were visualized and analysed using the ROVER software (ABX advanced biochemical compounds GmbH, Redeberg, Germany). Concentration curves were expressed as mean standardized uptake values (SUV).

### Data analysis

Statistical analysis was executed with GraphPad Prism software version 8.0 (GraphPad Software Inc., La Jolla, CA, USA). Flow cytometry data were analysed using FlowJo v.10 (FlowJo LLC, BD Life Sciences, Ashland, OR, USA).

## Supplementary information

Supplementary information.

## Data Availability

The datasets generated and/or analysed during the current study are available from the corresponding author on reasonable request.
