# Supplementary material for: “UniCAR”-modified off-the-shelf NK-92 cells for targeting of GD2-expressing tumour cells
Source: Sci Rep. 2020 Feb 7;10:2141. doi: 10.1038/s41598-020-59082-4 (PMC7005792; doi:10.1038/s41598-020-59082-4)
Supplement: Supplementary file 1 — Supplementary information. [file 41598_2020_59082_MOESM1_ESM.pdf]

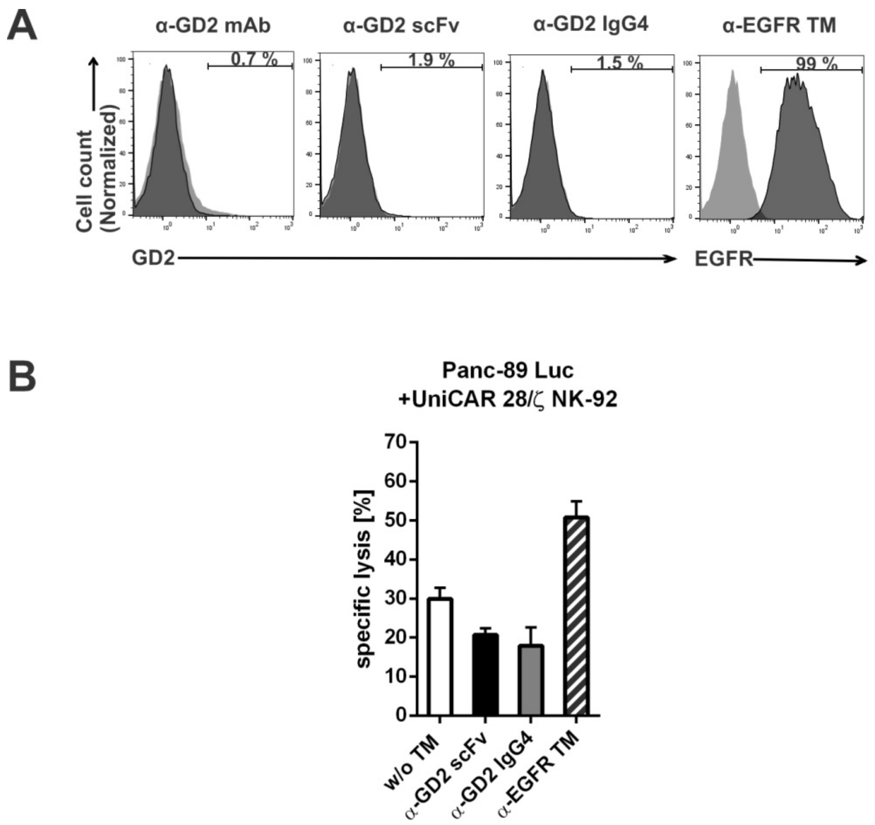

Supplementary Figure 1. Antigen-specific redirection of UniCAR NK-92 cells via α-GD2 TMs towards GD2-negative Panc-89 cells. (A) Panc-89 cells were stained with an α-GD2 mAb or an isotype-matched control Ab, followed by the secondary Ab (dark grey and light grey areas, respectively). Furthermore, Panc-89 Luc cells were incubated with α-GD2 scFv, α-GD2 IgG4 or α-EGFR TM (used as a positive control). TM binding was detected with anti-5B9 mAb specific for the E5B9 epitope tag, followed by Alexa Flour 647-conjugated goat α-mouse Ab (dark grey areas). As a control, cells were incubated with anti-5B9 mAb and the detection Ab without a TM (light grey areas). (B) UniCAR 28/ζ NK-92 cells were co-cultured at an E:T ratio of 5:1 with Panc-89 Luc cells in the presence or absence of α-GD2 scFv, α-GD2 IgG4 or α-EGFR TM (positive control) for 4 hrs. Thereafter, specific lysis was measured using a luminescence-based assay. Results are shown as mean ± SD of data from triplicates.
